# Regulation of biofilm formation by BpfA, BpfD, and BpfG in *Shewanella oneidensis*

**DOI:** 10.3389/fmicb.2015.00790

**Published:** 2015-08-04

**Authors:** Guangqi Zhou, Jie Yuan, Haichun Gao

**Affiliations:** Institute of Microbiology, College of Life Sciences, Zijingang Campus, Zhejiang UniversityHangzhou, China

**Keywords:** biofilm, *bpfA* bpfG, bpfD, regulation mechanism, *S. oneidensis*

## Abstract

Bacteria switch between two distinct life styles – planktonic (free living) and biofilm forming – in keeping with their ever-changing environment. Such switch involves sophisticated signaling and tight regulation, which provides a fascinating portal for studying gene function and orchestrated protein interactions. In this work, we investigated the molecular mechanism underlying biofilm formation in *Shewanella oneidensis* MR-1, an environmentally important model bacterium renowned for respiratory diversities, and uncovered a gene cluster coding for seven proteins involved in this process. The three key proteins, BpfA, BpfG, and BpfD, were studied in detail for the first time. BpfA directly participates in biofilm formation as extracellular “glue” BpfG is not only indispensable for BpfA export during biofilm forming but also functions to turn BpfA into active form for biofilm dispersing. BpfD regulates biofilm development by interacting with both BpfA and BpfG, likely in response to signal molecule c-di-GMP. In addition, we found that 1:1 stoichiometry between BpfD and BpfG is critical for biofilm formation. Furthermore, we demonstrated that a biofilm over-producing phenotype can be induced by C116S mutation but not loss of BpfG.

## Introduction

Biofilm is a type of surface-attached structure composed of microbial cells embedded in their self-produced extracellular polymeric substances (EPS), mainly exopolysaccharides, proteins, and extracellular DNA (Flemming and Wingender, 2010). It has been recognized as the principle life style for microbes in nature (O’Toole, 2003; Hall-Stoodley et al., 2004). Although planktonic cells are advantageous to look for favorable niches, biofilm allows cells to remain and thrive in such places. Thus, switching between planktonic and biofilm-forming modes represents a major life style change for microbes, and has been shown to be a tightly regulated process (O’Toole, 2003; Hall-Stoodley et al., 2004). Many regulatory cascades controlling transition of microbial life-styles studied to date involve regulatory factors to mediate transcription and translation of proteins for biosynthesis of EPS, including sigma factors, transcriptional factors, several nucleotide messengers, and sRNAs (Karatan and Watnick, 2009; Fazli et al., 2014).

**FIGURE 1:**
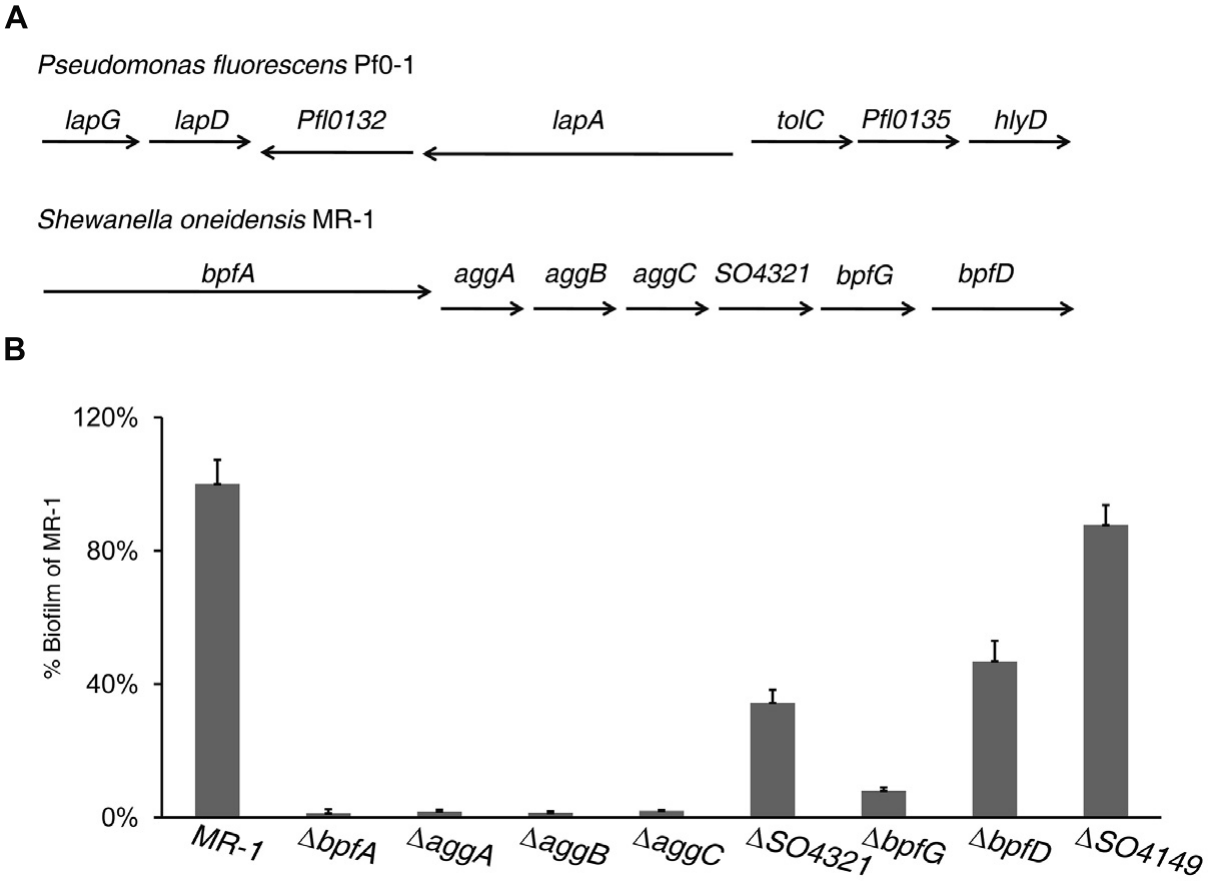
**Identification of a cluster of genes involved in biofilm formation in *Shewanella oneidensis* MR-1. (A)** Arrangement of genes in the *lapA* operon of *Pseudomonas fluorescens* Pf0–1 and their homologous in MR-1. The type I secretion system (TISS) is formed by ORF *tolC–Pfl0135–hlyD* in Pf0–1 and *aggA–C* (*SO4318–SO4320*) in MR-1. The ORFs *SO4321*, *SO4322* (*bpfG*), and *SO4323* (*bpfD*) encode an OmpA-like protein, a TISS associated periplasmic transglutaminase-like cysteine proteinase and a bifunctional diguanylate cyclase/phosphodiesterase protein, respectively. *Pfl0132* codes for a protein with no impact on biofilm formation. **(B)** Relative biofilm biomass of mutants lacking genes indicated. Biofilm formation was determined using the standard plate assay, and normalized to the value of MR-1 (wild-type) to yield the relative biofilm for comparison across different experiments. Error bar represents SE of three experiments.

However, there are exceptions. In *Pseudomonas fluorescens* Pf0–1, a protein network is reported to regulate biofilm development through sophisticated signaling and protein interactions rather than mediating EPS production (Newell et al., 2011). This system consists of multiple proteins encoded by genes in a *lap* cluster (**Figure 1A**). Of these Lap proteins, LapA, LapG, and LapD are critical to the process of biofilm formation. LapA, a Bap/RTX hybrid cell surface protein, is exported by a type I secretion system (TISS) encoded by three genes (*tolC*–*Pfl0135*–*hlyD*) immediately downstream of *lapA* and serves as a cell surface attached adhesin responsible for “gluing” cells together (El-Kirat-Chatel et al., 2014). LapG is a periplasmic proteinase, which cuts LapA off the outer membrane (Boyd et al., 2014). As a result, compared to the wild-type mutants lacking LapG are more robust in forming biofilm and overproduction of LapG promotes biofilm dispersion (Boyd et al., 2012). LapD is a transmembrane protein regulating LapG activity in response to signal molecule c-di-GMP (Newell et al., 2009). The environmental cue for the modification of LapA is conditions unfavorable for biofilm formation, such as low inorganic phosphate concentrations (Newell et al., 2011).

*Shewanella oneidensis* MR-1 is a Gram-negative facultative anaerobe, the best studied representative of the genus *Shewanella* (Venkateswaran et al., 1999). Following its isolation (Myers and Nealson, 1988), the bacterium was soon found to be able to use a wide range of solid electron acceptors (EA), a subset of which include minerals containing heavy metal ions such as Fe(III), Cr(VI), and U(VI), to name a few (Fredrickson et al., 2008). The feature renders MR-1 an appealing agent for bioremediation, developing microbial fuel cells (MFC) and synthesizing metal nanomaterials (Logan and Regan, 2006; Marshall et al., 2006; Pirbadian et al., 2014).

Previous studies of biofilm formation in MR-1 have identified BpfA (Bpf stands for Biofilm promoting factor) and AggA as essential proteins for the process (De Windt et al., 2006; Liang et al., 2010, 2012; Theunissen et al., 2010). Sequence comparison reveals that BpfA and AggA are analogous to *P. fluorescens* LapA and TolC, respectively (Boyd et al., 2014). Like LapA and TolC of *P. fluorescens*, BpfA and AggA are encoded by two genes next to each other, albeit oppositely oriented (**Figure 1A**). Additionally, AggA is the outer-membrane component of the accompanying TISS system (AggA–AggB–AggC), resembling TolC–Pfl0135–HlyD (Theunissen et al., 2009, 2010). Despite these similarities, the BpfA-mediated biofilm formation carries novel features as the low inorganic phosphate concentrations could not differentiate the *bpfA* mutant from the wild-type. Therefore, this work aims to further explore the BpfA-mediated biofilm formation in MR-1. Here, we first identified BpfA, BpfG, and BpfD to be the important players in biofilm development, through bioinformatic, mutational, and molecular analyses. Further investigation uncovered interactions between these proteins and their effects on biofilm formation and dispersion. Although these three proteins constitute a protein network vaguely resembling the one in *P. fluorescens*, mechanisms underlying roles played by each component differed significantly.

## Materials and Methods

### Strains and Growth Conditions

*Shewanella oneidensis* strains and *Escherichia coli* strains were cultured in LB medium at 30°C and 37°C, respectively. In detecting biofilm formation in high Pi and low Pi medium, the composition of medium is: low-phosphate (50 mM Tris-HCl, 0.2% Tryptone, lactate, 0.6 mM MgSO_4_); high-phosphate (add 1 mM K_2_HPO_4_ into low-phosphate medium). When needed, chemicals were added at the following concentrations: 2, 6-diaminopimelic acid, 0.3 mM; ampicillin, 50 μg/ml; gentamycin, 15 μg/ml; kanamycin, 50 μg/ml; and tetracycline, 12.5 μg/ml; chloramphenicol, 25 μg/ml; streptomycin, 12.5 μg/ml.

### Mutant Construction and Complementation

MR-1 in-frame deletion strains were constructed by the *att*-based Fusion PCR method (Jin et al., 2013). In brief, two fragments flanking the target gene were generated by PCR, with primers containing *attB* and gene specific sequences, and then were fused by a second round of PCR. The fusion fragments were introduced into plasmid pHGM01 by site-specific recombination using the BP Clonase (Invitrogen) according to the manufacturer’s instruction. The resulting mutagenesis vectors were transformed into *E. coli* WM3064, and the verified ones were transferred into proper MR-1 strains via conjugation. Integration of the mutated constructs into the chromosome were selected by resistance to gentamicin and confirmed by PCR. Verified *trans*-conjugants were grown in LB broth in the absence of NaCl and plated on LB supplemented with 10% sucrose. Gentamicin-sensitive and sucrose-resistant colonies were screened by PCR for deletion of the target gene. All mutations were verified by sequencing the mutated regions.

Plasmids pHG102 (Wu et al., 2011) and pHGE-P*_tac_* were used in genetic complementation of mutants. Genes of interest were amplified and inserted into MCS of pHG102 under the control of MR-1 *arcA* promoter, which is constitutively active (Gao et al., 2010) or pHGE-P*_tac_*, which is under IPTG induction (Luo et al., 2013). The resulting complementation vectors were transferred into the corresponding mutation strain via conjugation and their presence were confirmed by plasmid purification and restriction enzyme digestion.

pHGE-P*_tac_* containing *SO4322* (*bpfG*) or *SO4323* (*bpfD*) were used as the templates for site-directed mutagenesis with a QuikChange II XL site-directed mutagenesis kit (Stratagene) as described previously (Sun et al., 2013). Mutated PCR products were generated, subsequently digested by DpnI, and transformed into *E. coli* WM3064. After sequencing verification, the resulting plasmid was transferred into the MR-1 strains by conjugation.

To construct *bpfG* mutants lacking one of GGDEF, EAL, and HAMP domains, location of each domain was defined based on GenBank annotation and sequence alignment to characterized homologous proteins. The HAMP, GGDEF, and EAL domains are composed of residues from 174 to 221, from 238 to 383, and from 410 to 639, respectively. Mutants expressing truncated proteins (lacking one of the three domains) were constructed by the *att*-based Fusion PCR method described above.

### Biofilm Formation Assay and Quantification

Biofilm assays were carried out as described before (O’Toole and Kolter, 1998; Yuan et al., 2013), with some modifications. Briefly, an aliquot (20 μl) of an overnight culture grown in LB was transferred into 2 ml fresh LB medium contained in each well of a 24-well plate and grown with shaking at 250 rpm at 30°C for specified hours (or 8 h, if not specified). Biofilm quantification was conducted as following: Biofilm formed on the plate walls was stained using 0.25% crystal violet for 15 min at room temperature, rinsed with water and air-dried. Absorbed crystal violet was dissolved using 30% (v/v) acetic acid and transferred to a fresh flat bottom microtiter plate and the absorption of the solution at 540 nm was determined by microplate spectrophotometer. The wild-type was included in every plate to serve as an “internal standard” to control for batch-to-batch variation. Biofilm of all other strains were normalized to the value of the wild-type to yield the relative biofilm for comparison across different experiments. Readings from no less than three experiments per strain were used to calculate the average and SE.

### Promoter Activity Assay

Promoters for *bpfA* and the *SO4318–SO4323* operon were first predicted with bioformatics analysis. To construct the PbpfA-*lacZ* reporter, ∼400 bp DNA fragments upstream of the *bpfA* operon was amplified by PCR cloned into pTP327 (Shi et al., 2013). After verification by DNA sequencing, the reporter plasmid was transferred into each MR-1 strain by conjugation. Cell culture of targeted density was harvested by centrifugation. Cell pellets were washed once with PBS buffer, resuspended to an optical density of OD_600_ ≈ 1.0 and lysed. β-galactosidase activity assay was performed using an assay kit (Beyotime, China) as described previously (Wu et al., 2011).

### BpfA Extraction

Bacterial cultures were grown overnight in LB, and then sub-cultured into 40 ml of fresh LB at a 1:100 dilution and grown shaking at 250 rpm. After 6 h of incubation, cells were vortexed and harvested by centrifugation (12,000 *g*, 5 min). All resulting supernatant was passed through a 0.22 μM filter (Millipore, Billerica, MA, USA) to remove residual cells, and then concentrated in 100,000 MWCO filter column (Millipore, Billerica, MA, USA). The final volume in concentrator was the supernatant fraction. For membrane-anchored BpfA, cells were harvested as above; the pellet was washed once in 30 mM Tris-HCl (pH 8.1) and repelleted. Cell pellet were then resuspended in 20 ml 30 mM Tris-HCl-20% sucrose buffer, followed by the addition of 200 μl of 20 mg/ml lysozyme-0.1 mM EDTA (pH 7.3) and incubated on ice for 30 min. Following lysozyme treatment, 20 ml of 3 mM ETDA (pH 7.3) was added and the resulting extract was sonicated for 40 cycles (3 s sonication, 4 s interval). A 40 ml fraction of the extract was then centrifuged at 16,000 *g* for 60 min., the resulting pellet was resuspended in 500 μl of SDS sample buffer to yield the membrane fraction. Sample was boiled at 100°C for 5 min prior to SDS-PAGE display. Finally, 40 μl samples were displayed on 6 M urea-5% SDS gels.

### Bacterial Two-Hybrid Assay

A bacterial two-hybrid (B2H) system (BacterioMatch II Two-Hybrid System Vector Kit) was used to investigate protein–protein interactions *in vivo* in *E. coli* cells as described previously (Wu et al., 2011). Briefly, plasmid constructs were created by cloning the bait (DNAs for BpfG, BpfG_C116S_, BpfD, BpfD_peri_) and target (DNAs for BpfD and BpfA_Nterm_) into the pBT and pTRG vectors and verified by sequencing. BpfD_peri_ is the periplasmic domain (∼100 a.a.) of BpfD and BpfA_Nterm_ is the N terminus (∼200 a.a.) of BpfA. BpfA_Nterm_ was used since cloning of the entire BpfA was hindered by its large size and repetitive nature. As for BpfD_peri_, the rationale was that the transmembrane regions of full-length BpfD might be a problem and lead to the negative result between BpfG and BpfD in the B2H assay, and since BpfG locates in the periplasm, the periplasmic region of BpfD was used to probe BpfD and BpfG interaction. Moreover, because the N-terminal cytosolic domain of BpfD has only 39 a.a. (including signal peptide), we did not construct a fractional BpfD containing only this domain to probe its interaction with BpfA. We did not construct a BpfD The resulting plasmids were used to co-transform BacterioMatch II Validation Reporter competent cells on M9 salt agar plates containing 25 mg/ml chloramphenicol and 12.5 mg/ml tetracycline with or without 3-amino-1,2,4-triazole (3-AT). Plasmid pair of pBT-L/pTRG-G was used as the positive control, and Plasmid pair of pBT/pTRG was used as the negative control. The plates were incubated for 24 h at 37°C. If colonies are not apparent, transfer the plates to room temperature and continue to incubate the plates in a dark location (to preserve the tetracycline) for an additional 16 h. The second incubation may allow the growth of cells containing toxic proteins or weak interactors. Then, the positive interactions were confirmed by streaking colonies onto plates containing both 3-AT and streptomycin (12.5 mg/ml).

### Bioinformatics Analyses

Promoters were predicted with the program Neural Network (Reese, 2001). Membrane protein structure and domain functionality were predicted with TopPred and SMART, respectively (Claros and von Heijne, 1994; Letunic et al., 2015).

## Results

### The *SO4317*–*SO4323* Gene Cluster is involved in MR-1 Biofilm Formation

As shown in **Table 1** and **Figure 1A**, an *in silico* analysis revealed that two genes downstream of *bpfA* (*SO4317*), namely *SO4322* and *SO4323*, resemble *P. fluorescens lapG* and *lapD* genes in terms of sequence, predicted function, and gene arrangement in the seven-gene cluster *SO4317*–*SO4323*. To be consistent, we named *SO4322* and *SO4323* as *bpfG* and *bpfD*, respectively. To examine roles of these genes within the cluster in biofilm formation, we created in-frame deletion mutants for each gene. In agreement with previous reports (De Windt et al., 2006; Liang et al., 2010; Theunissen et al., 2010), BpfA and the TISS system encoded by *aggABC* genes (*SO4318–20*) were found to be crucial for biofilm formation in MR-1. Additionally, mutants missing *bpfG* or *bpfD* gene were also significantly defective in biofilm formation, indicating that both of the genes are required for the process (**Figure 1B**). Moreover, we found that *SO4321* (annotated to encode an OmpA family protein), the gene located between the TISS system coding genes and *bpfG*, had a role in biofilm formation as well (**Figure 1B**). Interestingly, MR-1 has another potential LapA homolog, SO4149, which may participate in Ca^2+^-mediated cell-cell adhesion because it contains a Cadherin repeat-like domain (Cao et al., 2005). However, impact of the SO4149 loss on biofilm formation was rather minor (**Figure 1B**). Given the essentiality of LapA to biofilm formation, a combination of the distinct phenotypes resulting from losses of BpfA and SO4149 and the syntenic similarity between *lapA* and *bpfA* concludes that BpfA is the counterpart of *P. fluorescens lapA*.

**Table 1:** BlastP results of *Pseudomonas fluorescens* LapA, LapG, and LapD against *Shewanella oneidensis.*

*P. fluorescens*	*S. oneidensis*	Annotation for *S. oneidensis* proteins	*E*-value
LapA	BpfA (SO4317)	Biofilm-promoting protein BpfA	2*e*–32
	SO4149	Secreted VCBS domain protein	4*e*–41
LapG	BpfG (SO4322)	Type I secretion system (TISS) associated periplasmic transglutaminase-like cysteine proteinase	3*e*–41
LapD	BpfD (SO4323)	Bifunctional diguanylate cyclase/phosphodiesterase LasD-like protein	3*e*–52

**FIGURE 2:**
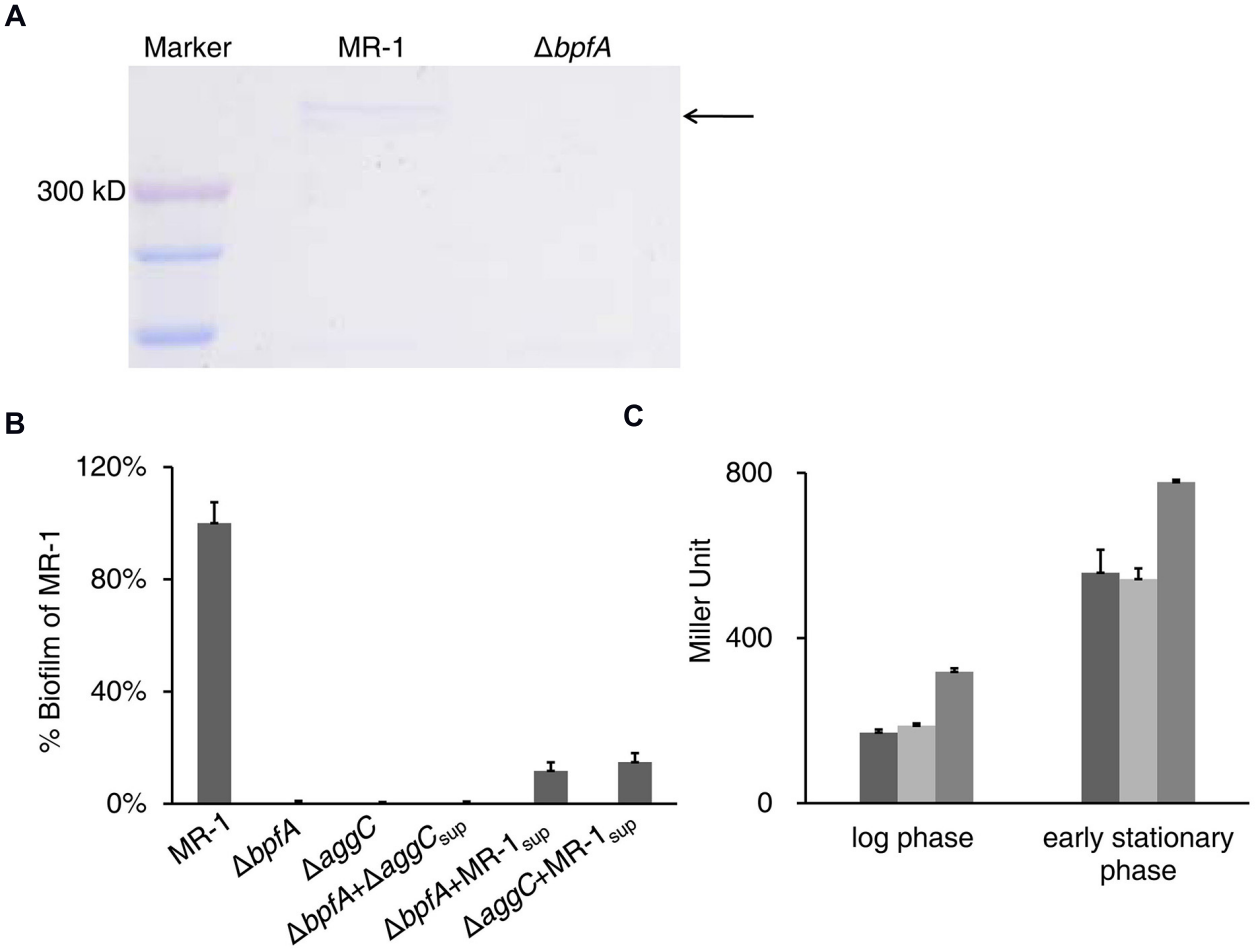
**BpfA is an extracellular adhesin required for biofilm formation. (A)** BpfA from culture supernatants of the indicated strains visualized by SDS-PAGE. Arrow indicates the band corresponding to BpfA protein. **(B)** Culture supernatant of wild-type could partially rescue the biofilm defect of Δ*bpfA* and Δ*aggC*. Supernatant prepared from wild-type or Δ*aggC* cells were added to the wells in a polystyrene plate containing LB-cultures of Δ*bpfA* or Δ*aggC*. Error bar represents SE of three experiments. **(C)** Promoter activity of *bpfA* in strains MR-1 (dark gray), Δ*bpfG* (light gray), or Δ*bpfD* (gray). The samples were collected in late log phase (OD_600_ ≈ 0.6) and early stationary phase (OD_600_ ≈ 1.0) for comparison. Error bar represents SE of three experiments.

BpfA possesses a Type I secretion C-terminal target domain *per* InterPro prediction and is believed to be exported by the TISS system coded by *aggABC* (De Windt et al., 2006; Theunissen et al., 2009). We found that BpfA protein can be collected from the supernatant of vigorously vortexed liquid cultures (**Figure 2A**), and such supernatant could partially rescue the biofilm defect of the BpfA mutant (**Figure 2B**). This observation supports the notion that BpfA is an outer-membrane adhesion. Moreover, BpfA promoter activity displayed a significant rise as cells enter the stationary phase, in good accordance with the expected timeline for biofilm development (**Figure 2C**).

The dual-band of BpfA in SDS-PAGE (**Figure 2A**) was intriguing, although not unusual for Bap family proteins (Cucarella et al., 2001). By excising and subjecting these bands to LC-MS/MS analysis, it was concluded that both bands arose from BpfA, and no perceivable post-translational modification was detected (Supplemental Tables S2 and S3). Efforts to clone *bpfA*, unfortunately, were hindered by its large size and repetitive nature. One interesting observation was that BpfA appears to be larger than the size deduced from the annotated genome sequence, both in terms of PCR product and SDS-PAGE result, which corresponded to each other well (Supplemental Figure S1). This is likely due to the sequencing error well known to be associated with genome regions rich in repeat sequences (Lesk, 2012). By resequencing, we confirmed 2038 bp from the translation initiation codon and 4167 bp ending at the stop codon. The remaining sequence between these two fragments is ∼2.6 kb longer than that reported in the genome sequence (2183 bp; Heidelberg et al., 2002). This fragment of ∼4.8 kb is composed of up to 16 300-bp repeats, based on sequence features of LapA (Boyd et al., 2014).

### BpfG is a Bifunctional Protein Necessary for BpfA Secretion and Cleavage

**FIGURE 3:**
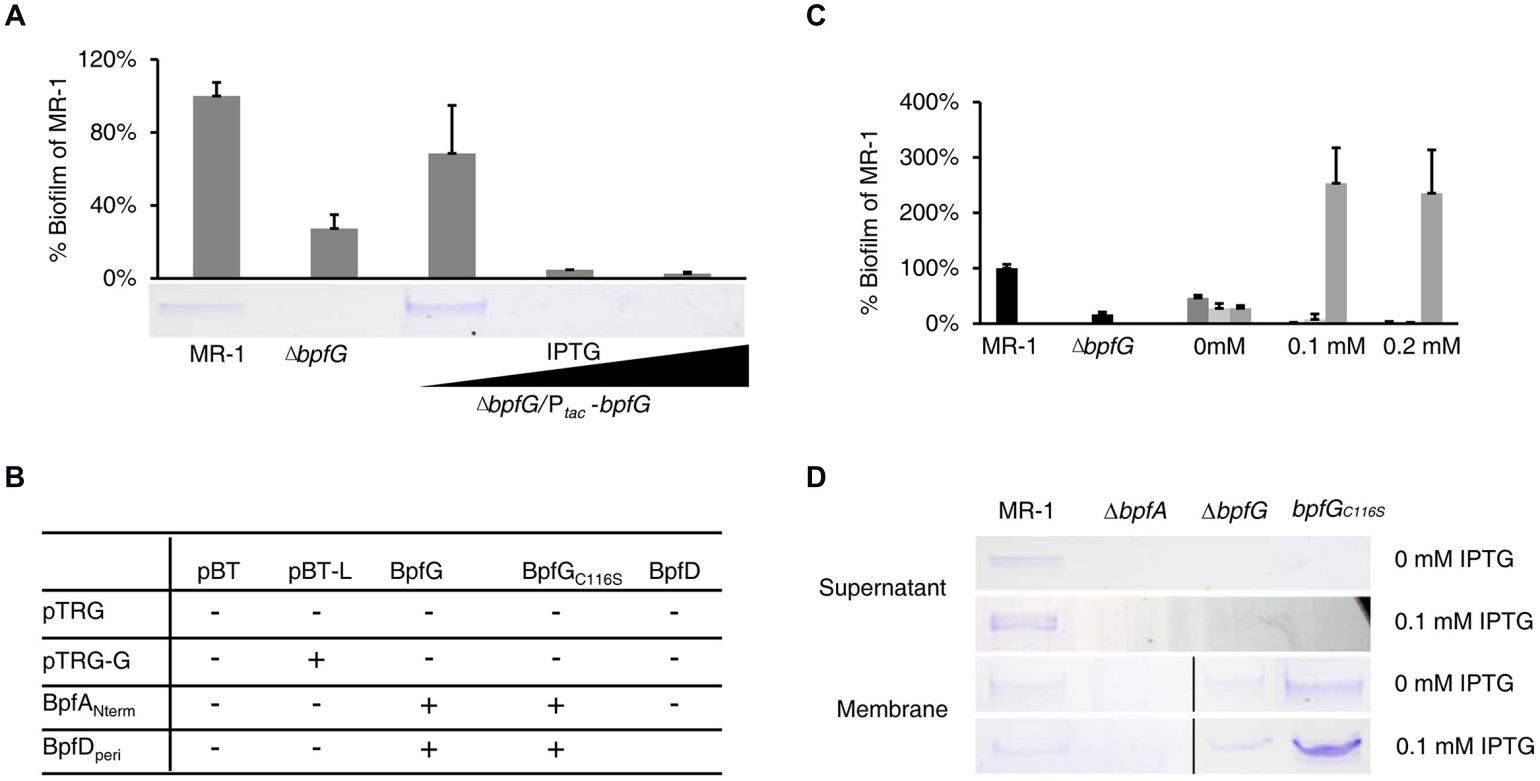
**BpfG is a periplasmic cysteine proteinase required for BpfA export and cleavage. (A)** The biphasic impact of BpfG on biofilm. Δ*bpfG* mutant was complemented with a plasmid carrying a P*_tac_*-*bpfG* construct, treated with different concentrations of IPTG (the black triangle underneath indicates the increase of IPTG level. 0, 0.1 mM, 0.2 mM from left to right, same in all figures unless specified otherwise). Error bar represents SE of three experiments. BpfA in the supernatant fraction were shown by protein electrophoresis. Arrow indicates the BpfA protein band. **(B)** Bacterial two-hybrid assay results of indicated proteins. “+” indicates positive results while “–” indicates that no interaction was detected in this assay. BpfA_Nterm_ is the N terminus (∼200 a.a.) of BpfA, and BpfD_peri_ is the periplasmic domain (∼100 a.a.) of BpfD, see Section “Materials and Methods” for more explanations. **(C)** Comparison of wild-type BpfG (dark gray) with BpfG_C15S_ (light gray) and BpfG_C116S_ (gray) in their ability to complement Δ*bpfD* for biofilm formation. The wild-type and Δ*bpfD* were provided for reference. P*_tac_* recombinant plasmids were treated with different concentrations of IPTG. Error bar represents SE of three experiments. BpfG_C18S_ and BpfG_C28S_ displayed similar phenotype as BpfG_C15S_ and were omitted in the figure for simplicity. **(D)** BpfA from the extracellular and membrane preparations of indicated strains were visualized by protein electrophoresis. Gels separated by a black line were from samples derived from the same experiment and processed in parallel.

As mentioned in the introduction, *P. fluorescens lapG* mutant (devoid of the periplasmic cysteine proteinase) displayed a biofilm-overproducing phenotype (Boyd et al., 2012). In contrast, deletion of *bpfG* led to a defect in MR-1 biofilm formation (**Figure 1B**), indicating a distinct role of BpfG compared with P. fluorescens LapG. Further analysis of the supernatant of vortexed Δ*bpfG* cultures revealed that BpfA was missing (**Figure 3A**), suggesting that BpfG influenced biofilm formation by modulating BpfA. Given that BpfG is annotated as a TISS associated periplasmic transglutaminase-like cysteine proteinase and located in the periplasm (Heidelberg et al., 2002; Brown et al., 2010), one possible scheme would be that BpfG is involved in certain process critical for BpfA excretion and function.

Interestingly, complementation of Δ*bpfG* with an IPTG-inducible P_tac_ plasmid revealed a biphasic effect of the protein on biofilm formation and BpfA (**Figure 3A**). More specifically, complementation in terms of biofilm formation and BpfA excretion was partially successful when no IPTG was added (the promoter was slightly leaky (Shi et al., 2014; Fu et al., 2015; Supplemental Figure S2), whereas IPTG at 0.1 mM or more abolished such effect, indicating that either missing or over-production of BpfG had negative impact on biofilm formation. By using the B2H assay, we detected direct interaction between BpfA and BpfG *in vivo* (**Figure 3B**). This finding, along with the fact that LapG modifies LapA in *P. fluorescens*, prompted us to hypothesize that BpfG might also be involved in BpfA cleavage, causing defect in biofilm formation when either in absence or over-abundance.

To provide evidence to support this hypothesis, we examined effects of the four cysteine residues of BpfG on biofilm formation, some of which are likely essential to the proteinase activity of BpfG. Plasmids expressing BpfG variants carrying C15S, C18S, C28S, and C116S mutations were individually introduced into the Δ*bpfG* strain and biofilm formation of resulting strains was examined. As shown in **Figure 3C**, phenotype of BpfG_C15S_, BpfG_C18S_, and BpfG_C28S_ were comparable to that of wild-type BpfG. However, overexpression of the BpfG_C116S_ mutant resulted in biofilm over-production. To our surprise, the amount of extracellular BpfA isolated in the supernatant of this mutant cultures was reduced to levels below the detection limit (**Figure 3D**), a scenario that was quite puzzling considering the established importance of BpfA in biofilm formation. Since BpfA most likely functioned as a cell-surface-attached extracellular adhesin, and the C116S mutation of BpfG did not block its interaction with BpfA (**Figure 3B**), we hypothesized that BpfG_C116S_ might change the nature of the association between BpfA and the cell membrane. SDS-PAGE analysis confirmed that BpfA isolated from the membrane fraction of the strain producing BpfG_C116S_ was more firmly associated with the cell membrane (**Figure 3D**). These results suggest that the 116th Cys residue is responsible for the observed negative impact of over-produced BpfG on biofilm formation, possibly by disrupting BpfA attachment to cell surface and even leading to degradation of unbounded BpfA. Taken together, these observations manifest that BpfG is a periplasmic cysteine proteinase that plays a role in both BpfA excretion and cleavage and the 116th residue is most likely to be the catalytic residue responsible for releasing BpfA from the membrane.

### BpfD Affects Biofilm Formation by Modulating BpfG Activity

**FIGURE 4:**
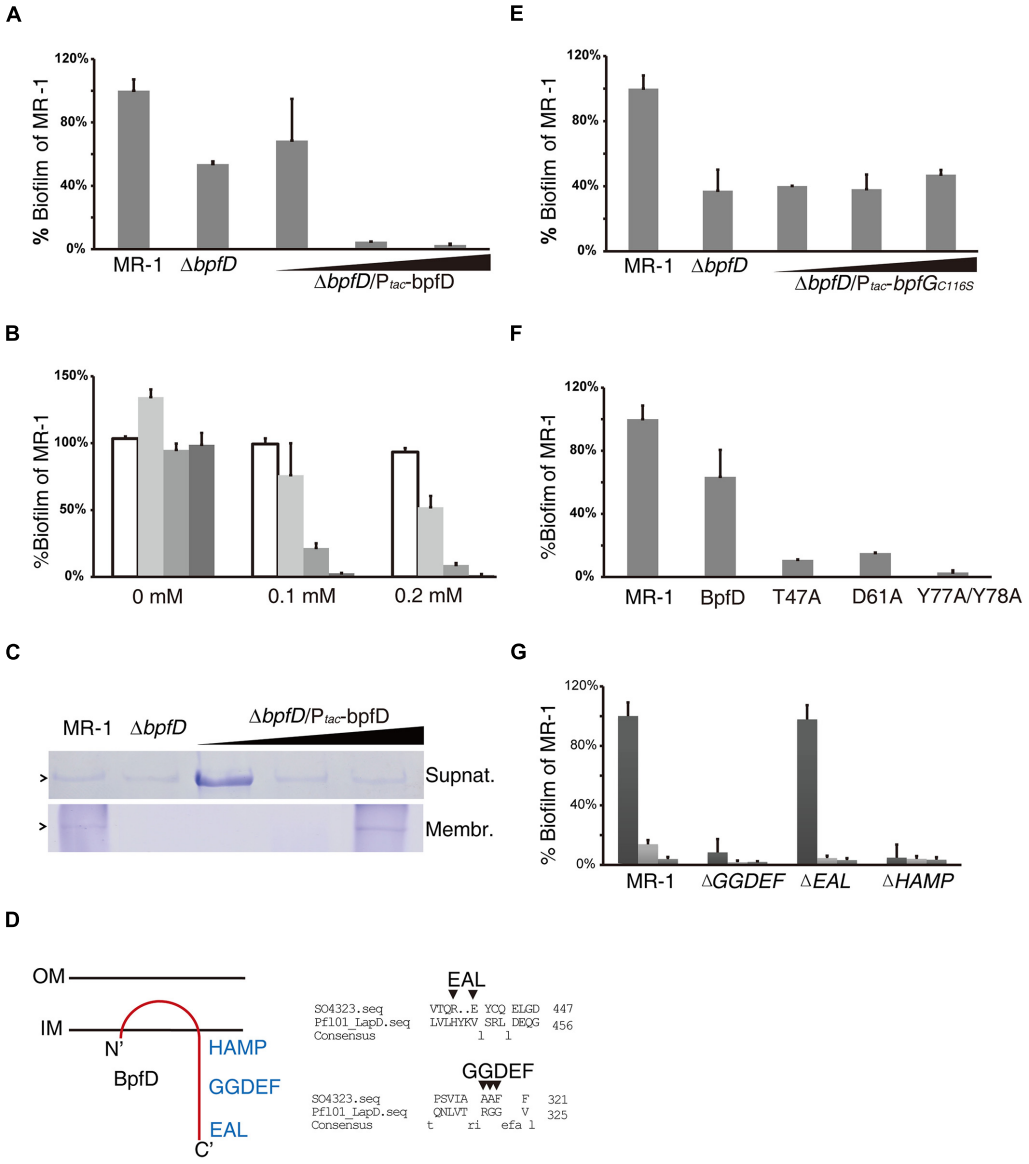
**BpfD is an inner membrane protein regulating biofilm formation by interacting with BpfG and BpfA. (A)** The biphasic impact of BpfD. Δ*bpfD* mutant was complemented with a plasmid carrying a P*_tac_*-*bpfD* construct, treated with different concentrations of IPTG. Error bar represents SE of three experiments. **(B)** A biofilm assay examining wild-type strain carrying P*_tac_*-GFP (white), Δ*bpfD*Δ*bpfG* strain carrying P*_tac_*-*bpfDbpfG* (light gray), P*_tac_*-*bpfD* (gray) and P*_tac_*-*bpfG* (dark gray). All P*_tac_* recombinant plasmids were treated with a range of concentrations of IPTG as indicated. Error bar represents SE of three experiments. **(C)** BpfA from the extracellular and membrane preparations were visualized by protein electrophoresis for the indicated strains, treated with different concentrations of IPTG. **(D)** Predicted orientation of BpfD (left), and alignment of BpfD and *P. fluorescens* LapD showing missing key residues as arrows indicate, numbers indicate amino acid position (right). **(E)** Over-expression of BpfG_C116S_ in ΔBpfD did not result in biofilm over-production. P*_tac_*-*bpfG*_C116S_ was treated with IPTG as indicated. **(F)** Point mutants BpfD_T47A_, BpfD_D61A_, and BpfD_Y 77AY 78A_ led to biofilm defect. Wild-type and mutant versions of BpfD were all carried individually on a P*_tac_* plasmid and assayed in Δ*bpfD* background with no IPTG added (the plasmid is leaky). **(G)** Biofilm formation of the wild-type, BpfD domain mutants Δ*GGDEF*, Δ*EAL*, and Δ*HAMP* for extended incubation (24 h: dark gray, 48 h: light gray, 72 h: gray).

Complementation of Δ*bpfD* with IPTG-induced *bpfD* expression displayed a biphasic impact similar to that of BpfG (**Figure 4A**). However, complementation of the Δ*bpfG*Δ*bpfD* double mutant with these two genes driven by one promoter was constantly successful (**Figure 4B**, light gray bars), indicating that a 1:1 stoichiometry between BpfG and BpfD may be crucial for biofilm formation. Furthermore, over-expression of either BpfG or BpfD in MR-1 undermined biofilm formation (**Figure 4B**); over-expression of GFP, on the other hand, did not have such impact, excluding the possibility of plasmid-related artifact (**Figure 4B**). One logical explanation for the observation would be that BpfG and BpfD interact with each other stoichiometrically, and their interaction is essential for regulating biofilm development. Moreover, we found that when BpfD in excess amounts of membrane-associated BpfA increased significantly, in addition to the biofilm defect (**Figure 4C**). Our interpretation of these data is that BpfD probably captures and holds BpfA to the cytoplasmic membrane, while interaction of BpfG and BpfD is required for BpfA release and export.

Consistent with this postulation, TopPred (Claros and von Heijne, 1994) predicted that BpfD is likely to be an inner-membrane protein encompassing a periplasmic domain close to the N-terminus, and a C-terminal cytosolic domain. In addition, the cytosolic domain might harbor a diguanylate cyclase GGDEF domain, a phosphodiesterase EAL domain, and a HAMP domain. SMART-based sequence analysis (Letunic et al., 2015) suggested that these domains are most likely degenerated since many key residues are absent (**Figure 4D**).

The predicted structure of BpfD, function of BpfG, and the resemblance of BpfG–BpfD to the *P. fluorescens* LapG–LapD system naturally led us to speculate that both systems may regulate biofilm formation through similar mechanisms. More specifically, BpfD may modulate BpfG activity (and thereby biofilm development) upon signals transmitted through its cytosolic domain. Consistent with this possibility, over-expression of BpfG_C116S_ in Δ*bpfD* mutant did not result in biofilm over-production as much as in Δ*bpfG* (**Figure 4E**), suggesting that BpfD probably regulates BpfG-mediated BpfA releasing from the membrane. A positive B2H assay result indicated that such modulation is achieved through direct interaction (**Figure 3B**). To confirm this, a semi-random point mutation screen was conducted to identify residues within BpfD important for the interaction. As shown in **Figure 4F**, when residue Thr^47^, Asp^61^, Tyr^77^, and Tyr^78^ were mutated to alanine (separately), amounts of biofilm reduced greatly. A comparative analysis of predicted tertiary structures for these BpfD variants revealed that none of these mutations altered protein configuration significantly (Supplemental Figure S3). Hence, the observed negative impact of the mutations on biofilm formation may be specific to the interaction between BpfG and BpfD.

To assess the function of the GGDEF, EAL, and HAMP domains of BpfD, mutants lacking each individual domain were constructed and their biofilm forming ability was evaluated. As shown in **Figure 4G**, the mutant lacking the EAL domain had capability to form biofilm comparable to the wild-type, whereas loss of either GGDEF or HAMP caused a defective phenotype. Additionally, dispersion of biofilm was also slower (judged from the reduction of bar height overtime in **Figure 4G**) in the latter two mutants as well as in the Δ*bpfD* strain, indicating that these domains might be important in regulating biofilm dispersion.

### Role of c-di-GMP

**FIGURE 5:**
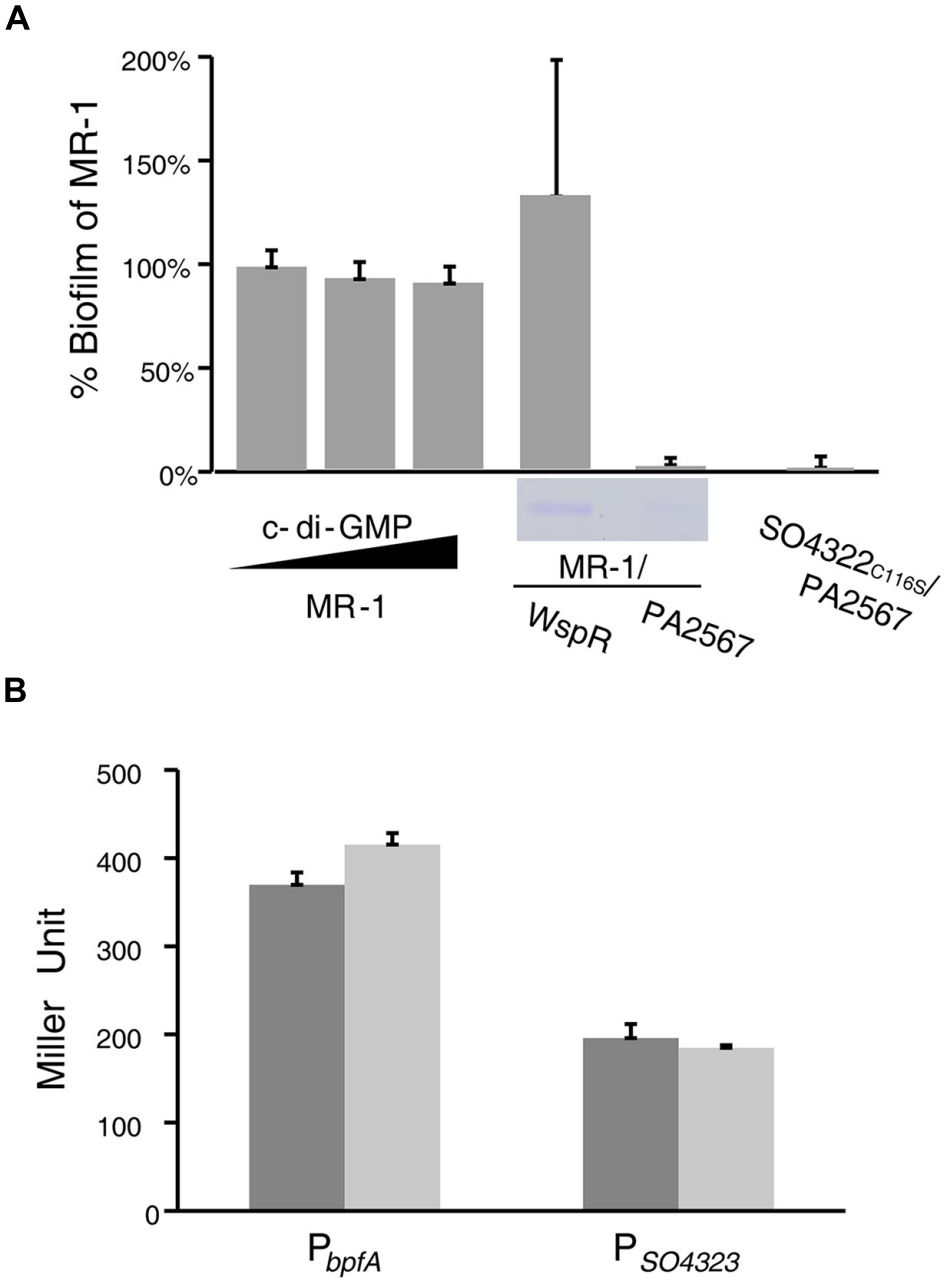
**MR-1 biofilm formation requires c-di-GMP but not sensitive to medium phosphate level. (A)** A biofilm assay examining MR-1, MR-1/P*_tac_*-WspR, MR-1/pHG102-PA2567, and BpfG_C116S_/pHG102-PA2567. MR-1/P*_tac_*-WspR was induced with 0.1 mM IPTG; MR-1 was treated with 0 mM, 60 mM and 120 mM c-di-GMP. BpfA in the supernatant fraction were shown by SDS-PAGE. **(B)** Promoter activities of *bpfA* and *bpfD* in MR-1 (dark) and MR-1/pHG102-PA2567 (light), respectively. The samples were collected in log phase (OD_600_ = 0.6).

Given the widely reported importance of c-di-GMP in biofilm formation, we examined the impact of this molecule in our system. As shown in **Figure 5A**, no significant change of biofilm production was observed through increasing c-di-GMP by either adding the purchased pure compound into the medium or over-producing WspR, a known *P. aeruginosa* diguanylate cyclase in the cell (De et al., 2008). Consistently, over-expression of PA2567, a verified phosphodiesterase from *P. aeruginosa* (Rao et al., 2009), abolished biofilm formation as well as extracellular presence of BpfA in both wild-type and the biofilm-overproducing BpfG_C116S_ strains.

Furthermore, the biofilm defect resulting from the *bpfD* mutation was not due to transcriptional suppression of the adhesin BpfA as expression of *bpfA* and *bpfG* did not change in MR-1 mutants overproducing phosphodiesterase PA2567 (**Figure 5B**). These data suggest that c-di-GMP is functionally associated with Bpf system but may not exert its impact as a transcriptional/translational cofactor.

## Discussion

Regulation of biofilm formation in microorganisms is a topic of both scientific and practical value. From the standpoint of basic scientific research, microbes have to stay tuned with the external environment to seize the cue(s) calling for the life-style switch from free-living to biofilm and make it through. Although how microorganisms make such decision remains largely unknown, it clearly involves orchestrated processes, including modulation of gene expression and interactions between proteins, as well as proteins and small molecules such as c-di-GMP (Karatan and Watnick, 2009). From a practical view point, biofilm has been associated with various problems in human daily life, especially in the food, environment, and biomedical fields (Leaper et al., 2010; Simões et al., 2010), better understanding of biofilm and its formation would help us tackle them.

Thanks to extensive studies on various microbes that have accumulated enormous amount of information, we now have gained considerable understanding of the numerous cellular networks that regulate biofilm formation. However, most of them concern regulation of EPS biosynthesis and other mechanisms are much less understood. In this work, we investigated roles of a protein cascade in biofilm formation in MR-1. We discovered that BpfA–BpfG–BpfD forms an interactive system that governs biofilm formation. Central to this system is BpfA, which is a very large protein although substantially smaller than *P. fluorescens* LapA. Because of a large number of repeats, the *bpfA* gene in the released genome sequence is ∼2.6 kb shorter. Each repeat, as illustrated in *lapA*, is about 300 bp for 100 amino acid residues (Boyd et al., 2014). Also because of these repeats, we failed to clone the full-length *bpfA* for complementation and other analyses, a situation encountered by O’Toole and Kolter (1998) team on *lapA* (personal communications). To date, many such proteins have been identified and exclusively function as surface-associated adhesions (Boyd et al., 2014).

**FIGURE 6:**
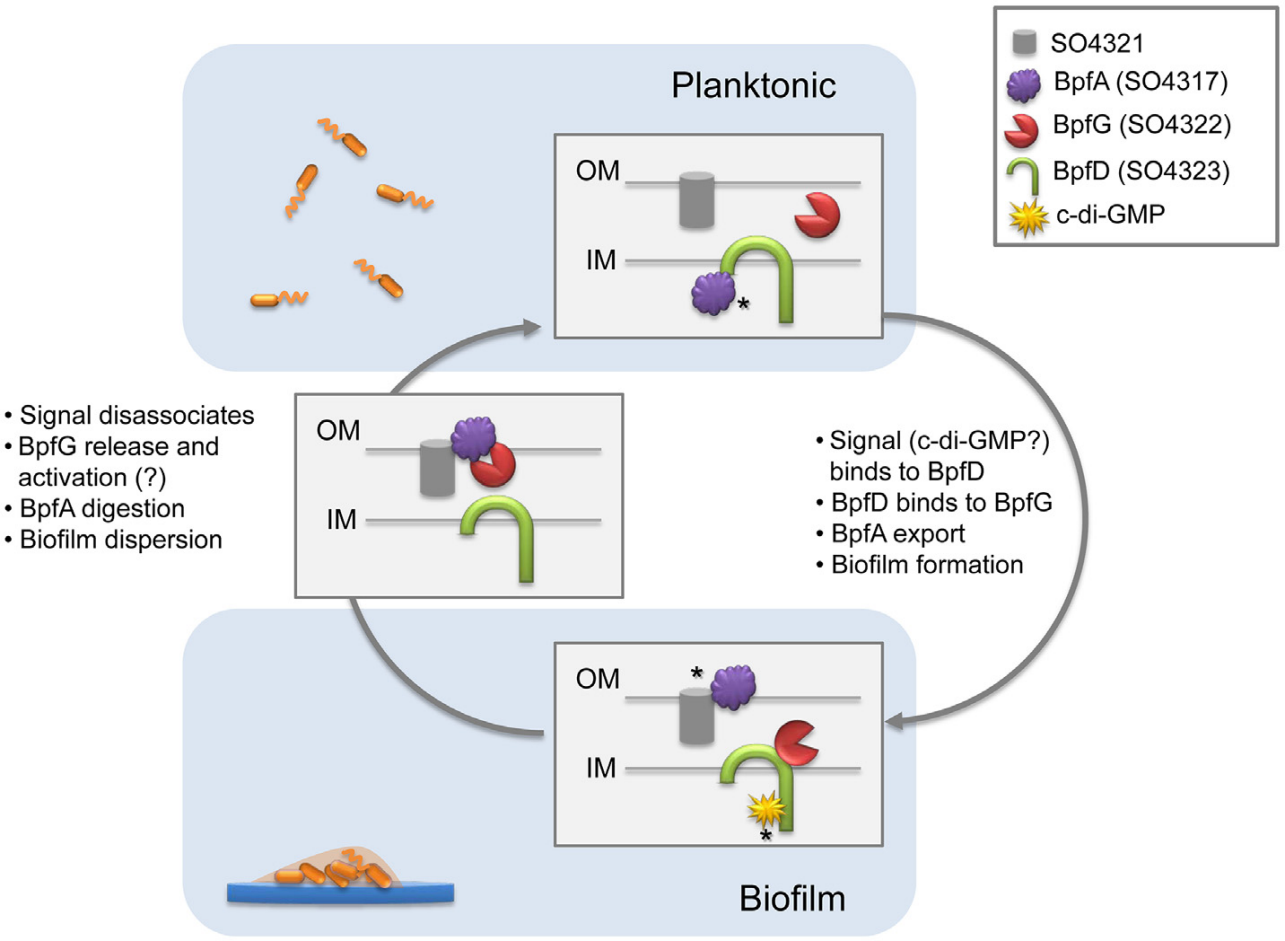
**Proposed model for regulatory biofilm formation mechanism in *S. oneidensis* MR-1**. In planktonic cells, BpfA is captured by BpfD to prevent biofilm formation. In biofilm forming stage, c-di-GMP and BpfG bind to BpfD, resulting in BpfA release and export by TISS system (not shown in figure). SO4321 facilitates BpfA localization on the outer membrane, and biofilm forms. c-di-GMP hydrolyzation and disassociation from BpfD leads to BpfG release and possibly activation of its proteinases activity, BpfA is subsequently digested and biofilm disperses. Asterisks in the figure indicate places of pure logical speculation at this point, and invites further investigation.

Although this three-membered system partially resembles LapA–LapG–LapD of *P. fluorescens* (Newell et al., 2011), there are significant differences in certain key features. In the best studied LapA–LapG–LapD system, the transmembrane receptor LapD is activated by high cytoplasmic concentrations of c-di-GMP, which in turn recruits the periplasmic protease LapG, preventing it from cleaving LapA, thereby promoting cell adhesion and subsequent biofilm formation (Newell et al., 2009, 2011; Boyd et al., 2012, 2014; Chatterjee et al., 2014). Our proposed model is as shown in **Figure 6**: BpfA, exported by the TISS channel formed by AggABC and anchored in the outer membrane, serves as a surface attached adhesin mediating cell–cell or cell–matrix adhesion. Our data suggest that intracellular BpfA molecules are probably held by BpfD before exportation, and free BpfA may not be exported efficiently. On the contrary, bindings of c-di-GMP and of BpfG to the respective cytosolic and the periplasmic domains of BpfD results in rapid BpfA exportation and biofilm formation. This explains the biphasic impact of BpfD on biofilm formation: enrichment of BpfA in the membrane fraction from cells overproducing BpfD and requirement of c-di-GMP and BpfG for biofilm formation. The balancing point is achieved when the stoichiometry between BpfG and BpfD is maintained at 1:1. BpfG may be released from BpfD upon c-di-GMP hydrolyzation or some other cues, which can move to BpfA for cleavage such that BpfA can be released from the outer membrane. When over-produced, BpfG overwhelms the BpfD control, leading to cleavage of BpfA and defect in biofilm formation. The role of the OmpA-like protein coded by *SO4321* in the process is not known. But given its location and predicted functions, the protein probably participates in translocation of BpfD and BpfG, as AggABC TISS transports protein from the cytosol to the extracellular space directly. Efforts to test this notion are under way.

Given the phylogenic closeness of *Shewanella* and *Pseudomonas* (Wu et al., 2008), it is not surprising that the “glue” function of BpfA, proteinase function of BpfG, and transmembrane regulator role of BpfD are shared between our model and the LapA–LapG–LapD network in *P. fluorescens*. However, the involvement of *SO4321* in biofilm formation, the essentiality of BpfG in BpfA export, and the critical 1:1 ratio of BpfG and BpfD are all data-supported novel aspects of the MR-1 Bpf system. In addition, the lack of impact of medium phosphate level on biofilm formation suggests that the upstream regulation is also distinct in *Shewanella*. It would be interesting to track down how BpfG and BpfD take on their new roles, as well as how the overarching regulatory scheme diverges in these closely related bacteria.

## Conflict of Interest Statement

The authors declare that the research was conducted in the absence of any commercial or financial relationships that could be construed as a potential conflict of interest.
