# Supplementary material for: Regulation of biofilm formation by BpfA, BpfD, and BpfG in Shewanella oneidensis
Source: Front Microbiol. 2015 Aug 4;6:790. doi: 10.3389/fmicb.2015.00790 (PMC4523816; doi:10.3389/fmicb.2015.00790)
Supplement: Supplementary file 1 [file Presentation_1.PDF]

Regulation of biofilm formation by BpfA, BpfD, and BpfG in *Shewanella oneidensis*

Guangqi Zhou, Jie Yuan*, Haichun Gao*

Author affiliation: Institute of Microbiology and College of Life Sciences, Zhejiang University, Hangzhou, Zhejiang, China

****Correspondence***:
Associate Professor Jie Yuan, jieyuan@zju.edu.cn
Professor Haichun Gao, haichung@zju.edu.cn
Institute of Microbiology and College of Life Sciences, Zhejiang University, Hangzhou, Zhejiang, 310058, China
Fax: +86-571-88981107

Supplemental Materials:

Supplemental Figure 1. PCR and SDS-PAGE analyses of *bpfA* and its product respectively

Supplemental Figure 2. The leakage of P_{tac} promoter

Supplemental Figure 3. BpfD of indicated point mutants may not significantly impact its structure

Supplemental Table 1. Strains and plasmid used in this study

Supplemental Table 2. BpfA sequences with MS detected segmented marked

Supplemental Table 3. List of BpfA segments detected in MS analysis

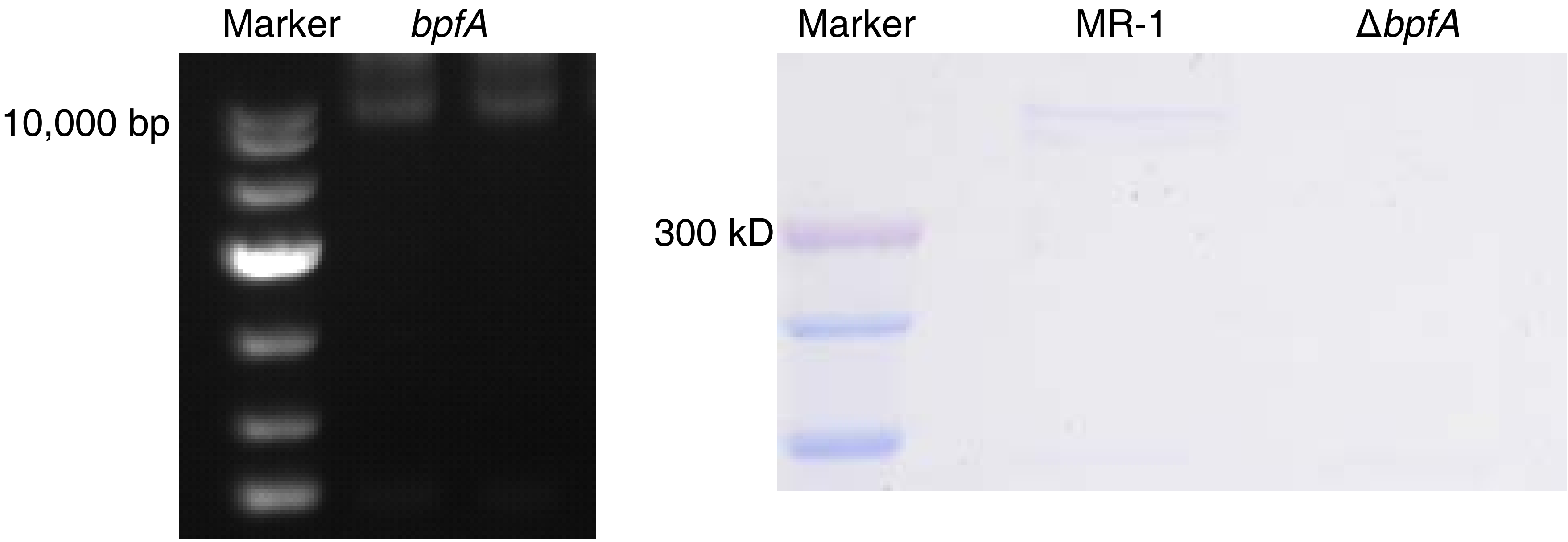

Supplemental Figure 1. *bpfA* PCR and SDS-PAGE bands.
The PCR product of *bpfA* gene was >10,000 bp,which was longer than the gene annotation in GenBank database (8,307 bp).

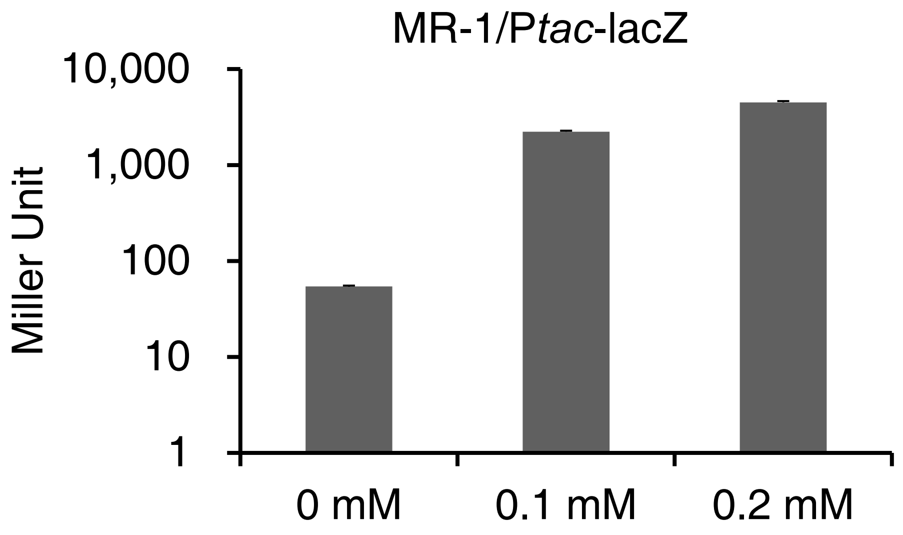

Supplemental Figure 2. P*tac* promoter leakage. A substantial amount of LacZ is expressed from the P*tac* promoter with no IPTG.

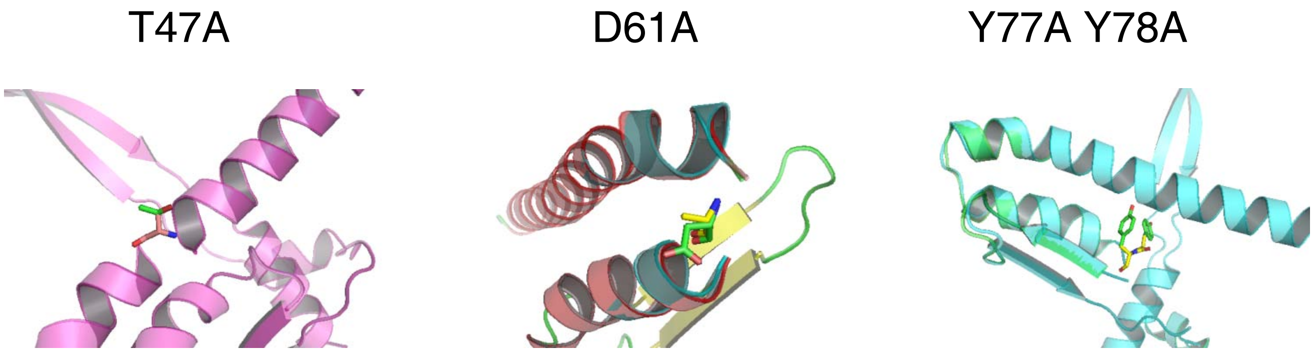

Supplemental Figure 3. BpfD point mutants did not change its protein structure. The structures of point mutants were predicted and overlayed with wild type BpfD by Pymol software.

Table S1. Strains and plasmids used in this study

Strain or plasmid	Description	Reference or source
Strain		
E. coli		
DH5α	Host for cloning	Lab stock
WM3064	Donor strain for conjugation, Δ*dapA*	W. Metcalf, UIUC
S. oneidensis		
MR-1	Wild type	Lab stock
HG0555	Δ*SO0555* derived from MR-1	This study
HG1558	Δ*SO1558* derived from MR-1	This study
HG1723	Δ*SO1723* derived from MR-1	This study
HG4149	Δ*SO4149* derived from MR-1	This study
HG4317	Δ*bpfA* derived from MR-1	This study
HG4318	Δ*aggC* derived from MR-1	This study
HG4319	Δ*aggB* derived from MR-1	This study
HG4320	Δ*aggA* derived from MR-1	Liang et al., 2010
HG4321	Δ*SO4321* derived from MR-1	This study
HG4322	Δ*bpfG* derived from MR-1	This study
$HG4322^{C116S}$	$BpfG^{C116S}$ derived from MR-1	This study
HG4323	Δ*bpfD* derived from MR-1	This study
HG4322-3	Δ*bpfG*Δ*bpfD* derived from MR-1	This study
$HG4323^{\Delta GGDEF}$	ΔGGDEF of BpfD derived from MR-1	This study
$HG4323^{\Delta EAL}$	ΔEAL of BpfD derived from MR-1	This study
$HG4323^{\Delta HAMP}$	ΔHAMP of BpfD derived from MR-1	This study
Plasmid		
pHGM01	Ap^r, Gm^r, Cm^r, *att*-based suicide vector	Jin et al., 2013
pHG102	pHG101 carrying *S. oneidensis* P_{arcA}	Wu et al., 2011
pHGE-P*tac*	Km^r, IPTG-inducible P_{tac} expression vector	Luo et al., 2013
pTP327	Broad host *lacZ* reporter vector	Gao et al., 2010
pBT	B2H vector	Stratagene
pTRG	B2H vector	Stratagene
pHG102-PA2567	Expression of *PA2567* under *S. oneidensis* P_{arcA}	This study
pHGE-P*tac-bpfG*	IPTG-inducible expression of *bpfG*	This study
pHGE-P*tac-bpfD*	IPTG-inducible expression of *bpfD*	This study
pHGE-P*tac-wspR*	IPTG-inducible expression of *wspR*	This study
pTP327-P_{bpfA}-*lacZ*	*lacZ* under control of P_{bpfA}	This study
pTP327-P_{bpfD}-*lacZ*	*lacZ* under control of P_{bpfD}	This study

Supplemental Table 2. BpfA sequence with peptides detected in MS analysis highlighted in yellow *

MGSVITSKKGLLKLVNGQINIEVDGSKQPAKDGEQLPKGAVLHIGENATYEITFDDG
TKLSNEVAPATEAMALPVTGEATPDEIQALQDLIASGEDPTTNLPETAAGNAPASDG
SSGYVSLARDGSESLATSGYSTSGQTLAAFATNSPEQSIATDSPSILANDSNTVAED
SVATGNVLNNDSDVDNELSVTSFTVSGQTVAAGTTVALEGGSLVINTDGSYTFTPNA
NWNGQVPVITYTTNTGSTATLTINVTPVDDPSVVVNDTNTVAEDSVATGNVLSNDSD
VDNELSVTSFTVSGQTVAAGTTVTLEGGSLVINTDGSYTFTPNANWNGQVPVITYTT
NTGSTATLTINVTPVDDASVLTNDSNTVAEDSVATGNVLSNDSDVDNELSVISFTVN
GQTVAAGTTVALEGGSLVINADGSYTFTPNANWNGQVPVITYTTNTGSTATLTINIT
PVANGGPSVTINTDTNNDGFISNEELGGATEVNVTIGLEGTGANTGDTLTVNGVDYI
LTQEDIDNGFVNLTLPAPAEGETITVVATITDAAGNTSPEGSDSAVLDTTGPVITVS
APDDTQDTTPTITGTTDAPPGSTITIVVTDSTGAQQTLTTTVNPDGTYAVDVTNPIA
EGGYTAEASVTDPAGNTGKASDNGNVDTKIDQDGDGNTVAITAITQDTGSSSSDFIT
NDNTLIFKGTVDLGDNSTLAVTINGVVYTTANGLVIDAQGNWSVDLTGTVLPDGIYP
VSATVTDVAGNSKTVTQDVVIDTKIDQDGDGNTVAITAITQDTGSSSSDFITNDNTL
IFKGTVDLGDNSTLAVTINGVVYTTANGLVIDAQGNWSVDLTGTVLPDGIYPVSATV
TDVAGNSKTVTQDVVIDTKIDQDGDGNTVAITAITQDTGSSSSDFITNDNTLIFKGT
VDLGDNSTLAVTINGVVYTTANGLVIDAQGNWSVDLTGTVLPDGTYPVSATVTDLAG
NSKTVTQDVLIDTQGPSVVVNIVDDVLTVGETSEVTFTFSEKVKDFEVGDLTMVGGT
VTDLKTTDGGKTWTGTFTPTPGFTGTASVTVNNGSYTDLNGNMGTGGQDTAPVDTQA
PSVVVNIVDDKLTVGETSEVTFTFSEKVKDFEVGDLTVVGGTVTDLKTTDGGKTWTG
TFTPTPGFTGTASVTVNNGSYTDLNGNVGTGGQDTAPVDTQAPSVVVNIVDDKLTVG
ETSEVTFTFSEKVKDFEVGDLTVVGGTVTDLKTTDGGKTWTGTFTPTPGFTGTASVT
VNNGSYTDLNGNVGTGGQDTAPVDTQAPSVVVNIVDDKLTVGETSEVTFTFSEKVKD
FEVGDLTVVGGTVTDLKTTDGGKTWTGTFTPTPGFTGTASVTVNNGSYTDLNGNVGT
GGQDTAPVDTQAPTAPTVLIVDDGTPGDGLLTQGEINSNGAGVQLTVSINAADFSAG
GHVNLTIVNGTATSNVELKLVNGELQFANGTPATGFTYNNGTISWTETAPANGQSIT
VTATQTDKAGNTSVQSTDTAIVYTPGNCNIVVNESTLRDGVPNIVSGTINFTAGTQA
LTAFNFNSSSINAATNLAGHNITWAIATNGALIGSINGVQAIILTLSDISAIAPGTS
GSITVNVELLDNLLQINGLNGENLSTLINGIVIEGTSVNGSVVTGNVNVEIVDDVPD
ANDDTGSVNVIVDSFRVSGVEANWTNWTNGTSVTTFDGNNNPNGGGTDNDSGLDQIR
WGNPINTYKSGYGFIDNDSALNGQFALNQDIILGTFTHYNFPISSGGAITKATMDIT
FSVTDAYGVVTPVTLKVNFDHNETPNNDNDPEASKDIIKVGNTNVTFEHQGQVYTLQ
VIGFRVPGTNQVVTEIKTAENAASSYELVVRIVAGDGYALPSTTGNVLFNDVIGADN
DLMVVGAAAGNQTGSGTSGNVGTIINGQYGTLIVFANGSYTYTVTANASAIPTGATE
TFTYTMQDADGDKSSALLTINVNTVNANALKAVQDQKNGPEDTAVIGNVLENDGNKN
TSVTHFTVANSATQHTAGSKITLAEGELTLNADGSYTFTPAADWNGQVPVITYTTNT
GATSTLTIVVTPVDDPTVTKPESKTIAEDALAKGNVLENDTDVDNTLSVTSFQVNGV
TYNAGNTWYQLPEGTLQLKTNGEYSFDPKDHWSGSLPVITYTTNTGATSTLNITVQA
VADAPNLTINGYTSVAAINFEDAKFSGSWDGVKANDIKGLNTIGTWHTSNRGGQVEI
GYESVYVSGGSNSNKVMEIEFNSGDKTLYTDIQADAGRFYELGFDIAARSGSVSTSG
LTIKLIPLDAQGNPLNAQAITLYDFDPTNANWLRDQKITLPINQSGKYRLQFEGDNG
DSYGALLDNLAFKVVDNMGYRGEFIKLSNISSSLKDTDTSETLSLKLQGLPEGAVLK

DALGNLATVGKDGTVDITNWDKSSLQIKVANHGNFTITVVATATETSNQDTAQSTAD
FQVTVLHPNNVVGGQSVDSFIMTNWTGNSAQFAVNLGGYGTVASSSKTEYIAKDTEL
LINAGNSNDYVDLGVSTANNIVNTGSSLPNLNNPVVTQAEILSSKFMAQDAITTNAG
TLKSDVLQPLQPKTDTVNLGSGDDTVNGGQGSQLVYGGSGDDLLIGGEGIDGLRGGD
GNDTLIGGLGDDVLRGDSGADTFVWRYADADKGTDHIMDFKVGEDKLDLSDLLQGET
ANTLESYLKFSLNNGSTVIDIDANKDGTFDQHIVLDGVNLYSQYGATNNAGIINGLL
GSNGQGPLIIDTQPTPVDLEVKLDPLKTVDPV

*: SDS-PAGE gel band shown in supplemental figure 1 was excised and subjected to trypsin digestion. The resulting peptides were analyzed by LC-MS/MS. Database search was performed using BioworksBrowser 3.3. For exact individual peptides detected, please refer to supplemental table 3.

Supplemental Table 3. List of BpfA segments detected in MS analysis

peptide detected in MS analysis	AA position in BpfA sequence
K.ATM*DITFSVTDAYGVVTPVTLK.V	1760-1784
K.AVQDQKNGPEDTAVIGNVLENDGNK.N	1968-1995
K.DALGNLATVGK.D	2393-2406
K.DALGNLATVGKDGTVDITNWDK.S	2393-2417
K.DFEVGDLTMVGGTVTDLK.T	1012-1032
K.DFEVGDLTVVGGTVTDLK.T	1111-1131, 1210-1230, 1309-1329
K.DGTVDITNWDK.S	2404-2417
K.DGTVDITNWDKSSLQIK.V	2402-2423
K.DTDTSETLSLK.L	2371-2384
K.FMAQDAITTNAGTLK.S	2552-2569
K.FSGSWDGVK.A	2189-2200
K.FSLNNGSTVIDIDANK.D	2687-2705
K.GLNTIGTWHTSNR.G	2203-2218
K.IDQDGDGNTVAITAITQDTGSSSSDFITNDNTLIFK.G	655-693,764-802, 873-911
K.LIPLDAQGNPLNAQAITLYDFDPTNANWLR.D	2283-2315
K.LQGLPEGAVLK.D	2382-2395
K.LSNISSSLK.D	2362-2373
K.LSNISSSLKDTDTSETLSLK.L	2362-2384
K.NGPEDTAVIGNVLENDGNK.N	1974-1995
K.NTSVTHFTVANSATQHTAGSK.I	1993-2016
K.SDVLQPLQPK.T	2567-2579
K.SGYGFIDNDSALNGQFALNQDIILGTFTHYNFPISSGGAITK.A	1718-1762
K.SSALLTINVNTVNANALK.A	1950-1970
K.TAENAASSYELVVR.I	1840-1856
K.TDTVNLGSGDDTVNGGQGSQLVYGGSGDDLLIGGEGIDGLR.G	2577-2620
K.TIAEDALAK.G	2075-2086
K.TLYTDIQADAGR.F	2248-2262
K.TNGEYSFDPK.D	2127-2139
K.TVTQDVVIDTK.I	753-766, 862-875
K.VGEDKLDLSDLLQGETANTLESYLK.F	2662-2689
K.VGNTNVTFEHQGQVYTLQVIGFR.V	1805-1830
K.VKDFEVGDLTMVGGTVTDLK.T	1010-1032
K.VKDFEVGDLTMVGGTVTDLKTTDGGK.T	1010-1038
K.VKDFEVGDLTVVGGTVTDLK.T	1109-1131, 1208-1230, 1307-1329
K.VKDFEVGDLTVVGGTVTDLKTTDGGK.T	1109-1137, 1208-1236, 1307-1335
K.VMEIEFNSGDK.T	2237-2250
K.VMEIEFNSGDKTLYTDIQADAGR.F	2237-2262
K.VNFDHNETPNNDNDPEASK.D	1782-1803
K.VNFDHNETPNNDNDPEASKDIIK.V	1782-1807
K.VVDNMGYR.G	2349-2359

R.FYELGFDIAAR.S	2260-2273
R.GDSGADTFVWR.Y	2636-2649
R.GGDGNDTLIGGLGDDVLR.G	2618-2638
R.GGQVEIGYESVYVSGGSNSNK.V	2216-2239
R.LQFEGDNGDSYGALLDNLAFK.V	2328-2351
R.SGSVSTSGLTIK.L	2271-2285
R.VPGTNQVVTEIK.T	1828-1842
R.WGNPINTYK.S	1709-1720
R.YADADKGTDHIMDFK.V	2647-2664
